# Supplementary material for: Transcriptomic-Proteomic Correlation in the Predation-Evoked Venom of the Cone Snail, Conus imperialis
Source: Mar Drugs. 2019 Mar 19;17(3):177. doi: 10.3390/md17030177 (PMC6471084; doi:10.3390/md17030177)
Supplement: Supplementary file 1 [file marinedrugs-17-00177-s001.pdf]

Transcriptomic-proteomic correlation in the predation-evoked venom of the cone snail, *Conus imperialis*

Ai-hua Jin[1]†, Sébastien Dutertre[2]†, Mriga Dutt[1], Vincent Lavergne[3], Alun Jones[1], Richard J. Lewis[1] and Paul F. Alewood[1]*

[1] Institute for Molecular Bioscience, The University of Queensland, St Lucia, Queensland 4072, Australia
[2] Institut des Biomolécules Max Mousseron, UMR 5247, Université Montpellier 2 - CNRS, Place Eugène Bataillon, 34095 Montpellier Cedex 5, France
[3] Léon Bérard Cancer Center, 28 rue Laennec, 69008 Lyon, France.

List of supporting information provided

Supplementary Table 1: 62 sequences common between two *C. imperialis* specimens.

Supplementary Table 2: Sequences validated by MS/MS.

Supplementary Table 3: Assembly of *C imperialis* venom duct for specimen 1 (S1) and specimen 2 (S2).

Supplementary Table 4: Comparison of assembled and raw data from two assemblers.

Supplementary Report: AGRF RNA quality measurement (2100 expert_mRNA Pico_DE54700697).

Supplementary Table 1: 62 sequences identified in both *C. imperialis* specimens.

**Name by superfamily**	**Precursor sequence**	**S1***	**S2***
A_1	MGMRMMFTVFLLVVLATTVVPITLASATDGRNAAADARMSPLISKFKKDYCHKYGYTIG	360	1689
A_2	MGMRMMFTVFLLVVLATTVVPITLASATDGRNAAANARVSPVISKFSKKWCHPNPYTVG	437	1665
A_3_Bn1.3	MGMRMMFTVFLLVVLATAVLPVTLDRASDGRNAAANAKTPRLIAPFIRDYCCHRGPCMVWCG	245	516
A_4	MGMRMMFTVFLLVVLATAVLPVTLDRASDGRNAAANAKTARLIAPFIRDYCCHRGPCMVWCG	2	526
A_5	MGMRMMFTVFLLVVLATTVVSITLDRASDGRNATVDNQLSALIGSPLSVWWKLHLLKHPLNHDKSPSA	52	47
A_6	MGMRMMFTVFLLVVLATTVVPITLASATDGRNAAADARMSPLISKFKRIIVISMVTLLGK	10	34
A_7	MGMRMMFTVFLLVVLATTVVPITLASATDGRNAAANARVSPVISKFSKNGVIRILTLWGK	9	28
A_8	MGMRMMFTVFLLVVLATTVVPITLASATDGRNAAADARMSPLISKFKKGLLS	3	16
A_9	MGMRMMFTVFLLVVLATTVVPITLASATDGRNAAANARVSPVISKFSKKWCHPNPYTVGK	2	14
A_10	MGMRMMFTVFLLVVLATTVVPITLASATDGRNAAANARVSPVISKFSRNGVIRILTLWGK	2	14
A_11	MGMRMMFTVFLLVVLATAVLPVTLDRASDEECSSQRQNASPDRAIHQGLLLS	2	9
A_12	MGMRMMFTVFLLVVLATTVVPITLASATDGRNAAADARMSPLISKLQKGLLS	2	6
A_13	MGMRMMFTVFLLVVLATTVVPITLASATDGRNAAADARMSPLISKFKKDYVISMVTLLGK	3	3
A_14	MGMRMMFTVFLLVVLATTVVPITLASATDGRNAAADARMSPLISKIQKRIIVISMVTLLGK	2	3
A_15	MGMRMMFTVFLLVVLATTVVPITLASATDGRNAAANARVSPVISKFSRNGVIRILTLWGNKAAAAAGPSEPRPAALCLTCFTFCLFVPLELNNLIH	2	3
D_1_Lt15.5	MPKLEMMLLVLLILPLCYIDAVGPPPPWNMEDEIIEHWQKLHCHEISDLTPWILCSPEPLCGGKGCCAQEVCDCSGPVCTCPPCL	438	1856
D_2	MPKLEMMLLVLLILPLCYIDAGPPPPWNMEDEIIEHWQKLHCHEISDLTPWILCSPEPLCGGKGCCAQEVCDCSGPVCTCPPCL	6	32
D_3	MPKLEMMLLVLLILPLCYIDAVGPPHHGTWKMKLLNIGKSFIVMKFQISHHGYCAARNHFAVEKAAVPRKSATALDQFVHARHVYSCSAHSPFSFPHDLALRKEPIKEQLQALRHI	4	12
D_4	MPKLEMMLLVLLILPLCYIDAVGPRHHGTWKMKLLNIGKSFIVMKFQISHHGYCAARNHFAVEKAAVPRKSATALDQFVHARHVYSCSAHSPFSFPHDLALRKEPIKEQLQALRHI	2	4
E_1	MMMRVFIAMFFLLALVEAGWPRLYDKNCKKNILRTYCSNKICGEATKNTNGELQCTMYCRCANGCFRGQYIDWPNQQTNLLFC	65	103
E_2	MMMRVFIAMFFLLALVEAGWPRLYDKNCRKTYCAPTAQIRYVERQRRIRMGNFSAPCTVGVQTAVFAVNTSIGPISRPISSSARPGFDHCLRRRERKWLM	7	12
E_3	MMMRVFIAMFFLLALVEAGWPRLYDKNCKKTYCAPTAQIRYVERQRRIRMGNFSAPCTVGVQTAVFAVNTSIGPISRPISSSARPGFDHCLRRRERKWLM	7	10
I1_1	MKLALTFLLILMILPLMTGEKTSDDLELRGVESLRAIFRDRRCSDNIGATCSDRFDCCGSMCCIGGQCVVTFAECS	6	28
I2_1	MFRVTSVLLVIVLLNLVVLTNACHMDCSKMTCCSGICCFYCGRPMCPGTRRALLQRLVGHQR	141	740
I2_2	MFRLTSVGCILLVIAFLNLVGLTNACTSEGYSCSSDSNCCKNVCCWNVCESHCRHPGKRTRLQGFFKHRR	161	257
I2_3	MFRVTSVLLVIVLLNLVVLTNACHMDCSKMTCCSGICCFTADVLCVLALGGRYSKD	17	85
I2_4	MFRLTSVGCILLVIAFLNLVGLTNACTSEGYSCSSDSNCCKMCAVGMYAKVTVDILERGRDSKDSLNIDVDMLPRGLLLFVVNGR	7	4
K_1_Im23b	MIMRMTLTLFVLVVMTAASASGDALTEAKRIPYCGQTGAECYSWCIKQDLSKDWCCDFVKTIARLPPAHICSQ	155	348
K_2_Im23a	MIMRMTLTLFVLVVMTAASASGDALTEAKRIPYCGQTGAECYSWCIKQDLSKDWCCDFVKDIRMNPPADKCP	105	174
K_3	MIMRMTLTLFVLVVMTAASASGDALTEAKRVPYCGQTGAECYSWCKEQHLIRCCDFVKYVGMNPPADKCR	60	109
K_4	MIMRMTLTLFVLVVMTAASASGDALTEAKRIPYCGQTGAECYSWCIKQDLSKDWCCDFVKDIRMNPRLTNVRNDCCSMP	12	12
K_5	MIMRMTLTLFVLVVMTAASASGDALTEAKRVPYCGQTGAECYSWCKEQHLIRCCDFVKYVGMNPRLTNVGNDCCSMP	7	11
K_6	MIMRMTLTLFVLVVMTAASASGDALTEAKRIPYCGQTGAECYSWCIKQDLSKAGVVISSRL	2	2
M_1	MMSTLVVLLTICLLMLPLTARQLDADQLADQLAERMEDISADQNRWFDPVKRCCMRPICMCPCCVNG	238	343
M_2_Eu3.3	MMSKLGVLLAICLLMLPLTALPLDGDQPQERKEDGKSAALQPWFDPVKRCCQAACSPWLCLPCCG	19	43
M_3_Bt3.1	MMSTLGVLLTIGLLLFPLTALPLDGDQPADQPAERLQDISPKEIPGSDPFKRCCHAPYCTPPHLGCPCCGK	41	25
M_4	MSKVGVVPLIFLVLLSIAALQNGDDPRRQRDEKQSPQGDILRSTLTKYSYNIQRRCWAGGSPCHLCSSSQVCIAPTGHPAIMCGRCVPILT	2	9
M_5	MSKVGVVPLIFLVLLSIAALQNGDDPRRQRDEKQSPQGDILRSTLTKYSYNIQRRCWAGAHPATCAAAVKFASRRLDTQQ	5	5
N_1	MSTLGMMLLILLLLVPLATFADDGPTMRGHRSAKLLAHTTRDSCPSGTNCPSKICCNGNCCSKSSCRCETNQATKERVCVC	16	6
O1_1_Conotoxin3	MKLRCMMIVAVLFLTASIFITADNSRNGIENLPRMRRHEMKKPKASKLNKRGCLPDEYFCGFSMIGALLCCSGWCLGICMT	6	18
O2_1_im6.2	MKLTILLLVAALLVLTQARTERRRVKSRKTSSTYDDEMATFCWSYWNEFQYSYPYTYVQPCLTLGKACTTNSDCCSKYCNTKMCKINWEG	188	481
O2_2	MKLTILLLVAALLVLTQARTERRRVKSRKTSSTYDDEMATFCGLIGMNFNIHIHIHMYNHALLWARLVRRTRIVVPSTVTPRCARLIGRDEPQTIRHHHSSLQRLQRLLFSV	3	17
O2_3	MEKLTILLLVTAVLMSTQALMQSGIEKRQRAKIKFFSKRKTTAERWWEGECYDWLRQCSSPAQCCSGNCGAHCKAW	8	9
P_1	MHLSLASSAALMLLLLFALGNFVGVQPGQIRDLNKGQLKDNRRNLQSQRKQMSLLKSLHDRNGCNGNTCSNSPCPNNCYCDTEDDCHPDRREH	38	56
P_2	MHLSTASSVALMFFLLFAFYGVQPELMTRDVDNGQLTDNRRNLRSRVKPTGLFKSRKPSEDCGKTCETAENCPDDCSSCLSVEGTYRCA	39	23
P_3	MHRSLAGSAVLMLLLLFALGNFVGVQPGLVTRDADNGQLMDNRRNLRLERKTMSLFKSLDKRADCSTYCFGMGICQSGCYCGPGHACMPNGR	15	20
P_4	MHLSLASSAALMLLLLFALGNFVGVQPGQIRDLNKGQLKDNRRNLQSQRKQMSLLKSLHDRNGCNGNTCSNTPLP	2	3
T_1_Im5.4	MRCLPVVVFLLLLLSAAAAPGVGSKTERLPGLTSSGDSDESLPFLNTICCWSGACCGG	494	1027
T_2	MCCIPVFFILLLLIPSAPSILAQPTTKGDVALASSYDDAKRTLQRLSIKYSCCPGIVSCCVIP	42	199
T_3	MRCLPVFIILLLLIPSAPSLTAQIKVKTNSSEASSHGNAKRTLQRLSTRIERCCPMEDRP	5	31
T_4	MRCLPVVVFLLLLLSAAAAPGVGSKTERLPGLTSSGDSDESLPFLNTILLLVRRLLWWLT	7	20
T_5	MRCLPVVVFLLLLLSAAAAPGVGSKTERLPGLTSSGDSDESLPFFEYNLLLVRRLLWWLT	16	19
T_6	MRCLPVVVFLLLLLSAAAAPGGRIENRKASRAHIIRRFRRESPLFEYNLLLVRRLLWWLT	2	7
T_7	MRCLPVVVFLLLLLSAAAAPGVGSKNRKASRAHIIRRFRRESPLFEYNLLLVRRLLWWLT	2	5
T_8	MRCLPVVVFLLLLLSAAAAPGVGSKTEGFQGSHHPEIPTRVSPFEYNLLLVRRLLWWLT	4	5
T_9	MRCLPVVVFLLLLLSAAAAPGSDRNRKASRAHIIRRFRRESPLFEYNLLLVRRLLWWLT	2	3
T_10	MRCLPVVVFLLLLLSAAAAPGVGSKPKASRAHIIRRFRRESPLLNTICCWSGACCGG	2	3
SF-im1_1	MARFLSILLCFAMATGLAAGIRYPDRVLGRCSTHDLSKMEIDTNLDGVYSPHRSFCTCGSGEVYFTAKDRRNHSNYRVYVCGMPTEFCTAENPVRDPKKGNRWLQCRCRQYKMVIYRDWLVLCE	7	25
SF-im2_1	MRLTTMHSVILMLLLVFAFDNVDGDEPGQTARDVDNRNFMSILRSEGKPVHFLRAIKKRDCTGQACTTGDNCPSECVCNEHHFCTGKCCYFLHA	14	31
SF-im3_1	MSKSGMLLFVLLLLLPLAIPELAPAGRSVTHHFRDFGAKRSVPISCVNPSTPNLQGSWQDKKCCSTKLCSPTNCCESSTCSCVEGSCQCL	4	55
SF-im4_1	MKFFTCLLLLLVVLTVVFDNVDACDRSCTGVMGHPSCATCCACFTSAGKRHADGQHSRMKVRTGAKNLLKRMPLH	5	27
SF-im5_1	MKTGMIICLLLIAFMDADGSPGDTLYSQKTADTDSGMKRFQKTFQKRRCVFCPKEPCCDGDQCMTAPGTGPFCG	2	10

*** The values in these couloumns refer to the read numbers of each paralog found in the transcriptomic analysis for each specimen**

Supplementary Table 2: Sequences validated by MSMS (99% confidence)

Name by superfamily	Seq number	MS/MS (> 99% conf)	Fixed modification	Enzyme Digestion	PTMs	Prec MW	z
A_3_Bn1.3	>im003	DYCCHR	Ethanolyl	Trypsin		883.34	2
D_1_Lt15.5	>im004	ILCSPEPLCGGK	Ethanolyl	Trypsin		1303.66	2
E_1	>im005	NTNGELQCTMYCR	Ethanolyl	Trypsin		1619.69	2
I1_1	>im006	CSDNIGATCSDR	Ethanolyl	Trypsin		1328.54	2
I2_1	>im007	MTCCSGICCFYCGR	Ethanolyl	Trypsin		1765.69	2
I2_2	>im008	NVCCWNVCESHCR	Ethanolyl	Trypsin		1727.7	3
K_1_Im23b	>im009	LPPAHICSQ	Ethanolyl	Trypsin		1008.52	2
K_2_Im23a	>im010	MNPPADKCP	Ethanolyl	Trypsin		1015.47	2
K_3	>im011	YVGMNPPADK		Trypsin		1090.54	2
M_1	>im012	CCMRPICMCPCCVN	Ethanolyl	Trypsin	Hyp@10, Amidated@C-term	1853.85	2
M_4	>im015	CWAGGSPCHLCSSSQVCIAPTGHPAIMCGR	Ethanolyl	Trypsin	Hyp@7, HyP@20	2181	3
N_1	>im016	ICCNGNCCSKSSCR	Ethanolyl	Trypsin		1724.69	2
O2_1_im6.2	>im018	ACTTNSDCCSK	Ethanolyl	Trypsin		1263.49	2
O2_3	>im019	QCSSPAQCCSGNCGAHCK	Ethanolyl	Trypsin	Hyp@5	2018.78	3
P_3	>im022	ADCSTYCFGMGICQSGCYCGPGHACMPN	Ethanolyl	Trypsin	M(oxi)@26, Amidated@C-term	3154.23	3
T_1_Im5.4	>im023	FLNTICCWSGACCG	Ethanolyl	Trypsin	Amidated@C-term	1651.79	2
SF-im1_1	>im025	VYVCGMPTEFCTAENPVRDPK	Ethanolyl	Trypsin		2443.33	2
SF-im2_1	>im026	EHHFCTGK	Ethanolyl	Trypsin	PyroGlu@N-term	983.45	2
SF-im4_1	>im028	CDRSCTGVMGHPSCATCCACFTSA	Ethanolyl	Trypsin	Hyp@12, Amidated@C-term	2689.09	3
SF-im5_1	>im029	CVFCPK	Ethanolyl	Trypsin		783.37	2
SF-im6_1	>im031	TGECDKNPGYMVNH	Ethanolyl	Trypsin		1607.68	3

Supplementary Table 3: Assembly of *C imperialis* venom duct for specimen 1 (S1) and specimen 2 (S2).

**IMP S1 (Total De-multiplexed 249,349 and total assembled 198,889)**				
	Raw Reads	***Newbler Contigs***	***Newbler Isotigs***	***Trinity***
# Sequences	220,516	703	473	1,594
Length Interval	24-770	1-3,060	58-3,060	101-3,075
Average Length	396.70	436.81	709.57	481.38
N50	459	700	770	502
N75	504	1,197	1,301	818
N90	528	1,690	1,723	1,387
%GC	52.41	47.67	47.04	46.30
%N	0	0	0	0
Total # bases	87,478,648	307,076	335,626	767,323

**IMP S2 (Total De-multiplexed 379,998 and total assembled 281,952)**				
	Raw Reads	***Newbler Contigs***	***Newbler Isotigs***	***Trinity***
# Sequences	332,390	2,256	1,386	6,021
Length Interval	22-737	1-6,389	26-6,389	101-6,994
Average Length	393.38	463.77	816.63	497.22
N50	459	824	953	521
N75	503	1,382	1,619	868
N90	527	2,218	2,513	1,428
%GC	50.62	46.59	46.36	45.34
%N	0	0	0	0
Total # bases	130,754,372	1,046,266	1,131,845	2,993,735

Supplementary Table 4: Comparison of assembled raw data from two assemblers. Detected sequences (Y) and undetected sequences (N) have been highlighted along with their subsequent detection in the MSMS spectra.

	Newbler assmebly		Trinity assembly		Raw reads		
**Name by superfamily**	**S1**	**S2**	**S1**	**S2**	**S1**	**S2**	**MSMS**
**A_1**	N	N	Y	Y	360	1689	
**A_2**	Y	N	N	N	437	1665	
**A_3_Bn1.3**	Y	N	N	Y	245	516	Y
**A_4**	N	N	N	N	2	526	
**A_5**	Y	N	N	N	52	47	
**A_6**	N	N	N	N	10	34	
**A_7**	N	N	N	N	9	28	
**A_8**	N	N	N	N	3	16	
**A_9**	N	N	N	N	2	14	
**A_10**	N	N	N	N	2	14	
**A_11**	N	N	N	N	2	9	
**A_12**	N	N	N	N	2	6	
**A_13**	N	N	N	N	3	3	
**A_14**	N	N	N	N	2	3	
**A_15**	N	N	N	N	2	3	
**D_1_Lt15.5**	Y	Y	Y	Y	438	1856	Y
**D_2**	N	N	N	N	6	32	
**D_3**	N	N	N	N	4	12	
**D_4**	N	N	N	N	2	4	
**E_1**	Y	Y	Y	N	65	103	Y
**E_2**	N	N	N	N	7	12	
**E_3**	N	N	N	N	7	10	
**I1_1**	Y	Y	Y	Y	6	28	Y
**I2_1**	Y	Y	Y	Y	141	740	Y
**I2_2**	N	N	Y	Y	161	257	Y
**I2_3**	N	N	N	N	17	85	
**I2_4**	N	N	N	N	7	4	
**K_1_Im23b**	Y	Y	Y	Y	155	348	Y
**K_2_Im23a**	Y	Y	N	N	105	174	Y
**K_3**	Y	Y	N	N	60	109	Y
**K_4**	N	N	N	N	12	12	
**K_5**	N	N	N	N	7	11	
**K_6**	N	N	N	N	2	2	
**M_1**	Y	Y	Y	Y	238	343	Y
**M_2_Eu3.3**	N	Y	N	N	19	43	
**M_3_Bt3.1**	N	Y	N	N	41	25	
**M_4**	N	N	N	Y	2	9	Y
**M_5**	N	N	N	N	5	5	
**N_1**	Y	N	Y	N	16	6	Y
**O1_1_Conotoxin3**	N	N	N	Y	6	18	
**O2_1_im6.2**	Y	Y	Y	Y	188	481	Y
**O2_2**	N	N	N	N	3	17	
**O2_3**	Y	N	Y	Y	8	9	Y
**P_1**	Y	Y	Y	Y	38	56	
**P_2**	N	N	Y	N	39	23	
**P_3**	Y	Y	Y	Y	15	20	Y
**P_4**	N	N	N	N	2	3	
**T_1_Im5.4**	Y	N	Y	Y	494	1027	Y
**T_2**	N	N	Y	N	42	199	
**T_3**	N	Y	N	N	5	31	
**T_4**	N	N	N	N	7	20	
**T_5**	N	N	N	N	16	19	
**T_6**	N	N	N	N	2	7	
**T_7**	N	N	N	N	2	5	
**T_8**	N	N	N	N	4	5	
**T_9**	N	N	N	N	2	3	
**T_10**	N	N	N	N	2	3	
**SF-im1_1**	Y	N	N	N	7	25	Y
**SF-im2_1**	N	N	N	Y	14	31	Y
**SF-im3_1**	N	Y	Y	Y	4	55	
**SF-im4_1**	N	N	N	N	5	27	Y
**SF-im5_1**	N	Y	N	Y	2	10	Y
	18	16	15	18			
**S2 only**							

S_1	N	N	N	N	0	10	
SF-im6_1	N	Y	N	N	0	6	Y
**S1 only**							
T-**S1**	N	N	N	N	41	0	Y

Note: Absent sequences after assembly had been unambiguously confirmed by MSMS, highlighting a major issue with current assembly algorithms, as this approach can eliminate valid sequences.

Electropherogram Summary

Ladder

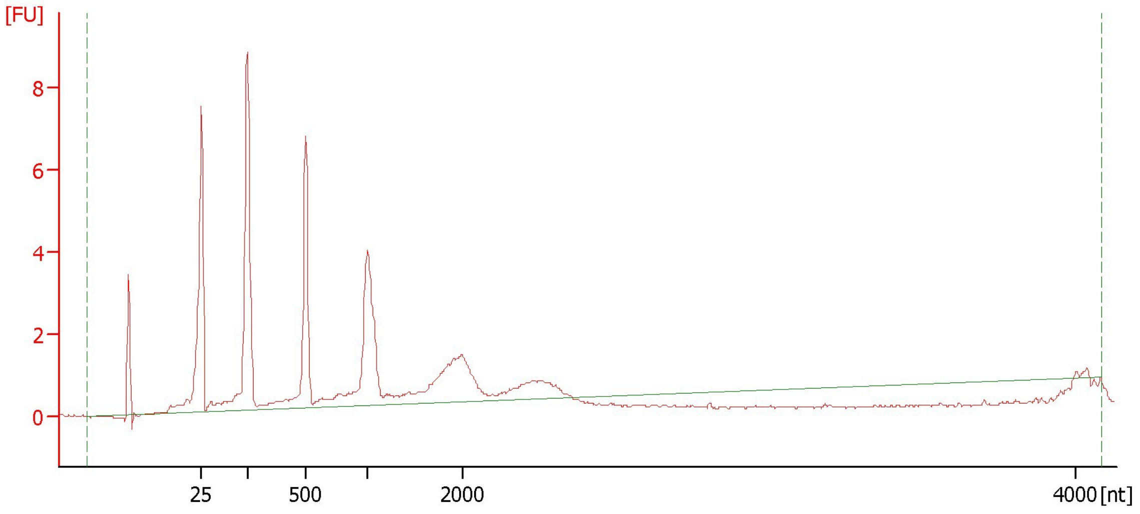

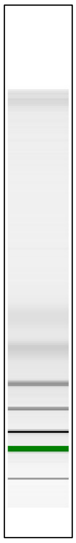

Overall Results for Ladder

RNA Area:	52.0	RNA Concentration:	1,000 pg/µl

 © Copyright 2003 - 2009 Agilent Technologies, Inc.

Assay Class:	mRNA Pico	Created:	1/16/2013 2:44:32 PM
Data Path:	C:\...16\2100 expert_mRNA Pico_DE54700697_2013-01-16_14-44-32.xad	Modified:	1/16/2013 3:08:43 PM

Electropherogram Summary Continued ...

SDIMPSMA - 1:10

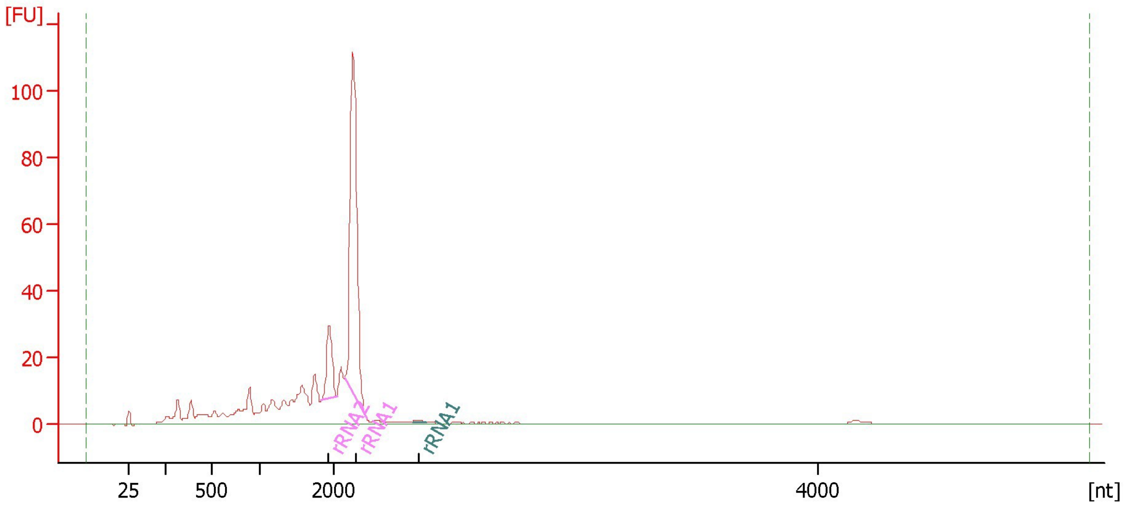

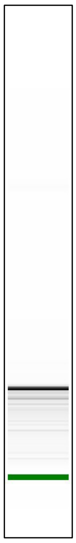

Overall Results for sample 3 : SDIMPSMA -1:10

RNA Area:	498.8	rRNA Contamination:	38.4 %
RNA Concentration:	9,600 pg/µl		

Fragment table for sample 3 : SDIMPSMA -1:10

Name	Start Size [nt]	End Size [nt]	Area	% of total Area
rRNA2	1,830	2,011	29.7	6.0
rRNA1	2,045	2,136	161.0	32.3
rRNA1	2,324	2,379	0.6	0.1

 © Copyright 2003 - 2009 Agilent Technologies, Inc.

Assay Class:	mRNA Pico	Created:	1/16/2013 2:44:32 PM
Data Path:	C:\...16\2100 expert_mRNA Pico_DE54700697_2013-01-16_14-44-32.xad	Modified:	1/16/2013 3:08:43 PM

Electropherogram Summary Continued ...

SDIMPMED - 1:10

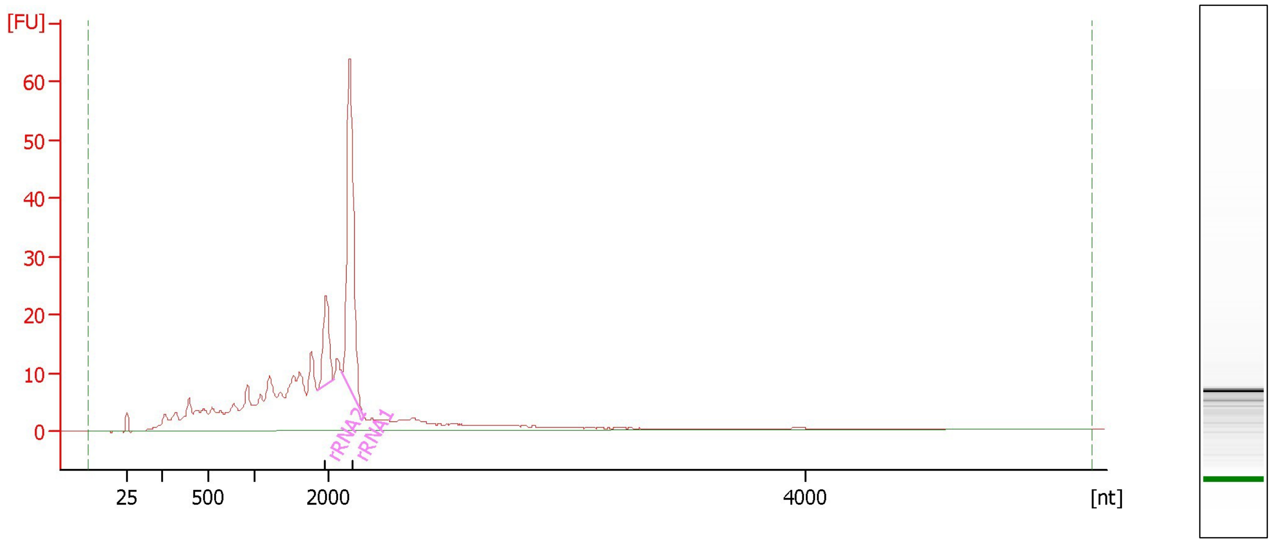

Overall Results for sample 4 : SDIMPMED-1:10

RNA Area:	435.2	rRNA Contamination:	24.9 %
RNA Concentration:	8,377 pg/µl		

Fragment table for sample 4 : SDIMPMED-1:10

Name	Start Size [nt]	End Size [nt]	Area	% of total Area
rRNA2	1,862	2,022	22.3	5.1
rRNA1	2,056	2,146	85.9	19.7

 © Copyright 2003 - 2009 Agilent Technologies, Inc.
